# The suppression of ghrelin signaling mitigates age-associated thermogenic impairment

**DOI:** 10.18632/aging.100706

**Published:** 2014-12-15

**Authors:** Ligen Lin, Jong Han Lee, Odelia Y. N. Bongmba, Xiaojun Ma, Xiongwei Zhu, David Sheikh-Hamad, Yuxiang Sun

**Affiliations:** ^1^ USDA/ARS Children's Nutrition Research Center, Department of Pediatrics, Baylor College of Medicine, Houston, TX 77030, USA; ^2^ State Key Laboratory of Quality Research in Chinese Medicine, Institute of Chinese Medical Sciences, University of Macau, Macao, China; ^3^ Department of Endocrinology, The first Affiliated Hospital of Zhengzhou University, Zhengzhou, 450052, China; ^4^ Department of Pathology, Case Western Reserve University, Cleveland, Ohio 44106, USA; ^5^ Department of Medicine, Baylor College of Medicine, Houston, TX 77030, USA; ^6^ Huffington Center on Aging; Department of Molecular and Cellular Biology, Baylor College of Medicine, Houston, TX 77030, USA

**Keywords:** ghrelin, growth hormone secretagogue receptor (GHSR), brown adipose tissue (BAT), thermogenesis, insulin signaling, mitochondrial biogenesis and dynamics

## Abstract

Aging is associated with severe thermogenic impairment, which contributes to obesity and diabetes in aging. We previously reported that ablation of the ghrelin receptor, growth hormone secretagogue receptor (GHS-R), attenuates age-associated obesity and insulin resistance. Ghrelin and obestatin are derived from the same preproghrelin gene. Here we showed that in brown adipocytes, ghrelin decreases the expression of thermogenic regulator but obestatin increases it, thus showing the opposite effects. We also found that during aging, plasma ghrelin and GHS-R expression in brown adipose tissue (BAT) are increased, but plasma obestatin is unchanged. Increased plasma ghrelin and unchanged obestatin during aging may lead to an imbalance of thermogenic regulation, which may in turn exacerbate thermogenic impairment in aging. Moreover, we found that GHS-R ablation activates thermogenic signaling, enhances insulin activation, increases mitochondrial biogenesis, and improves mitochondrial dynamics of BAT. In addition, we detected increased norepinephrine in the circulation, and observed that GHS-R knockdown in brown adipocytes directly stimulates thermogenic activity, suggesting that GHS-R regulates thermogenesis via both central and peripheral mechanisms. Collectively, our studies demonstrate that ghrelin signaling is an important thermogenic regulator in aging. Antagonists of GHS-R may serve as unique anti-obesity agents, combating obesity by activating thermogenesis.

## INTRODUCTION

Obesity and diabetes have reached epidemic proportions in all age groups, but are most pronounced among the elderly [1, 2]. Obesity is characterized by increased fat mass and dysfunction of adipose tissues. There are 2 types of adipose tissues: white adipose tissue (WAT) is a specialized lipid storage organ for storing excess calories; in contrast, brown adipose tissue (BAT) contains large amounts of mitochondria and uses lipids to generate heat [3, 4]. Upon cold-stimulus, the sympathetic nervous system (SNS) is stimulated, which releases norepinephrine (NE) into BAT to activate β3-adrenergic receptor (β3-AR). Subsequently, uncoupling protein 1 (UCP1) is recruited into mitochondria, which promotes lipolysis and heat production [3, 4]. Non-shivering thermogenesis in BAT was recently recognized to play a crucial role in energy balance in rodents and human neonates [3, 5-7]. Thermogenic activity of BAT is positively correlated with energy expenditure, and dysregulation of thermogenesis in BAT is linked to obesity in humans [6]. Most recently, studies have shown that enhanced thermogenesis also improves glucose homeostasis and insulin sensitivity in animals [8] and humans [9]. These results suggest that interventions to increase BAT mass and/or activity may be very attractive strategies for prevention/treatment of obesity and diabetes.

Aging is associated with severe thermogenic impairment; BAT declines 95% in mass and 75% in activity in old men, as compared to young men [10, 11]. Thermogenic impairment likely contributes to age-associated obesity [12], However, the factors underlying the dysfunction of BAT in aging are currently unknown. During aging, cumulative molecular damage leads to impairment and functional decline [13, 14]. Age-associated mitochondrial dysfunction is involved in pathogenesis of metabolic disorders and neuro-degenerative diseases [15-18]. Mitochondria are dynamic organelles responsible for cellular energy production in response to cell signals [19]. Mitochondrial dynamics are determined by fusion and fission processes; the balance between fusion and fission is essential for the maintenance of normal mitochondrial function. Fusion is a ‘joining event’ between two different mitochondria, mediated by mitofusins (Mfns) and optic atrophy gene 1 (OPA1); fission is a process dividing one mitochondrion into two mitochondria, mediated by dynamin-related protein 1 (*Drp1*) and fission 1 (*Fis1*) protein [20]. It was recently reported that expression of *Mfn2* and *Drp1* genes is reduced in the skeletal muscle of elderly humans [21], and smaller and fragmented mitochondria are more abundant in the muscle of obese and type 2 diabetic subjects [22]. Furthermore, reduced expression of fission-related protein *Drp1* has been linked to decreased mitochondrial complex IV activity in HeLa cells [23]. *Drp1* deletion has been shown to decrease mtDNA content [24], and cause brain developmental defects and severe neuro-degeneration [25].

Ghrelin is the only known orexigenic hormone to increase appetite and promote obesity [26-28]. We and others have reported that ghrelin's effects on GH release and appetite are mediated through its receptor, the Growth Hormone Secretagogue Receptor (GHS-R) [29-31]. Ghrelin is ubiquitously expressed, but the highest level of expression is detected in the stomach and intestine [32]. The expression of GHS-R is more restricted; the highest expression is detected in pituitary and brain, but lower levels of expression are detectable in some peripheral tissues, including WAT and BAT [31, 33, 34]. It has been reported that ghrelin stimulates lipid accumulation in WAT [35, 36], but suppresses norepinephrine (NE) release of BAT [37, 38]. Aging is associated with insulin resistance, and ghrelin is known to increase insulin resistance [39]. We have found that deletion of *Ghsr* gene enhances BAT thermogenesis in aged mice, resulting in a lean and insulin-sensitive phenotype [34].

Ghrelin (*aka* “active ghrelin”), and its related peptides des-acyl ghrelin (DAG) and obestatin, are all derived from the same preproghrelin gene [40, 41]. However, only ghrelin activates GHS-R; the effects of DAG and obestatin are mediated through other receptors [40]. We have shown that DAG has ghrelin-like effects on feeding, but it cannot activate GHS-R [42]. It has also been shown that DAG stimulates lipid accumulation in visceral fat [35], has adipogenic effects in bone marrow [43], and prevents diet-induced adipose inflammation and development of diabetes [44]. In contrast, obestatin is an anorexic hormone; its effect on appetite is opposite from ghrelin [41]. We previously reported that *ghrelin*-null nice (*Ghrelin^−/−^*), absent of ghrelin, DAG and obestatin, show no thermogenic phenotype [45]. On the other hand, *Ghsr* knockout mice (*Ghsr^−/−^*, where ghrelin's effect is blocked but the effects of DAG and obestatin remain intact) show enhanced thermogenesis [34]. The differential thermogenic phenotypes of *Ghrelin^−/−^* and *Ghsr^−/−^* mice raise the question of whether DAG and/or obestatin have opposing effects on thermogenesis as compared to ghrelin.

To better understand the role of ghrelin signaling in the regulation of age-associated decline of thermogenesis, we analyzed ghrelin and its related peptides in young, middle-aged and old mice, and characterized thermogenic signaling cascades, insulin activation, and mitochondrial biogenesis and dynamics in BAT of old *Ghsr^−/−^* mice. We also investigated whether GHS-R mediated thermogenesis is likely regulated centrally and/or peripherally.

## RESULTS

### Obestatin increases UCP1 expression in HIB1B cells, but DAG has no effect

**Figure 1:**
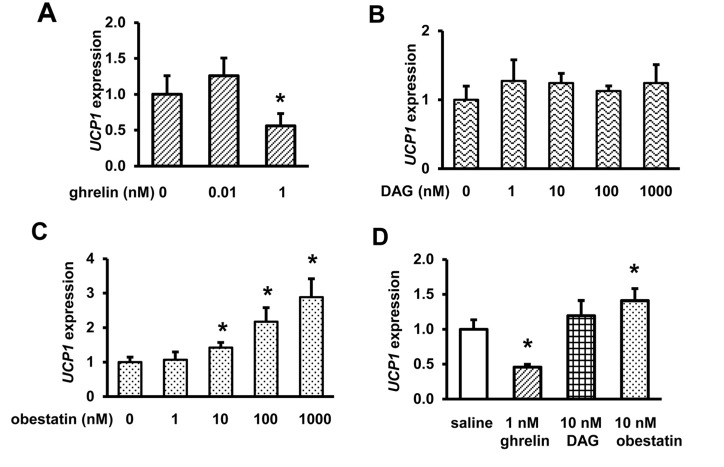
Ghrelin and obestatin exhibit differential effects on *UCP1* expression in differentiated brown adipocyte HIB1B cells *UCP1* expression in HIB1B cells treated with saline or different concentrations of ghrelin (**A**), des-acyl ghrelin, DAG (**B**), and obestatin (**C**). Summary of *UCP1* expression in HIB1B cells treated with saline, 1 nM ghrelin, 10 nM DAG, or 10 nM obestatin (**D**). *N* = 9, and each assay was measured in triplicate. *p<0.05, Treatments vs. Controls.

Our previous study showed that ghrelin inhibits adipogenesis and suppresses *UCP1* expression in brown adipocyte HIB1B cells [34]. DAG and obestatin have been reported to have different physiological effects from ghrelin in several cell types and tissues [40, 41, 46, 47]. To assess the effects of DAG and obestatin on thermogenesis, we treated differentiated HIB1B cells with different concentrations of DAG or obestatin. Consistent with our previous report [34], 1 nM ghrelin inhibits *UCP1* expression in differentiated HIB1B cells (Fig. 1A). In contrast, DAG had no effect on *UCP1* expression even at high concentration (Fig. 1B), but obestatin increased *UCP1* gene expression in a dose-dependent manner (Fig. 1C). Ghrelin and obestatin showed opposite effects on *UCP1* expression in brown adipocytes (Fig. 1D), suggesting that ghrelin and obestatin may have opposing effects on thermogenesis.

### Circulating ghrelin is increased in aging, but DAG and obestatin are not changed

**Figure 2:**
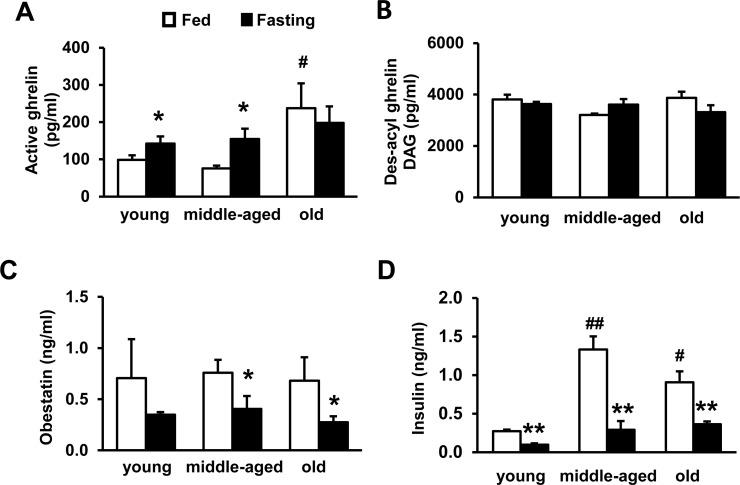
Plasma concentrations of active ghrelin, des-acylated ghrelin, and obestatin during aging Active ghrelin (**A**), des-acyl ghrelin, DAG (**B**), obestatin (**C**), and insulin (**D**) of young, middle-aged and old WT mice under fed or fasting conditions. Young (4-months), middle-aged (10-months) and old (22-months) mice were used. The mice were fasted overnight. *N* = 10-12. *p<0.05, fed vs. fasting. #*p*<0.05, ## P<0.001, young vs. middle-aged or old.

To assess whether ghrelin, DAG and obestatin regulate thermogenesis and insulin resistance during aging, we measured circulating ghrelin, DAG, obestatin and insulin concentrations in young, middle-aged and old wild-type (WT) mice, under either fed or fasting conditions. Under fed condition, ghrelin concentration in old mice was significantly higher than young and middle-aged mice (Fig. 2A). Interestingly, fasting only increased ghrelin in young and middle-aged mice, but not old mice (Fig. 2A), suggesting that ghrelin regulation in old mice is less responsive to fasting. The concentrations of DAG and obestatin didn't change during aging (Fig. 2B and 2C); while fasting did not affect DAG (Fig. 2B), fasting significantly decreased obestatin in middle-aged and old mice (Fig. 2C). Elevated ghrelin in aging increases lipolysis and exacerbates insulin resistance [39]. Consistently, our data showed that fed insulin levels were higher in middle-aged and old mice relative to young mice, and fasting reduced insulin (Fig. 2D). The data support that increased ghrelin in old mice promotes age-associated insulin resistance, which may further impair BAT function in aging.

### GHS-R ablation attenuates age-associated decline of thermogenesis and activates insulin signaling in BAT

**Figure 3:**
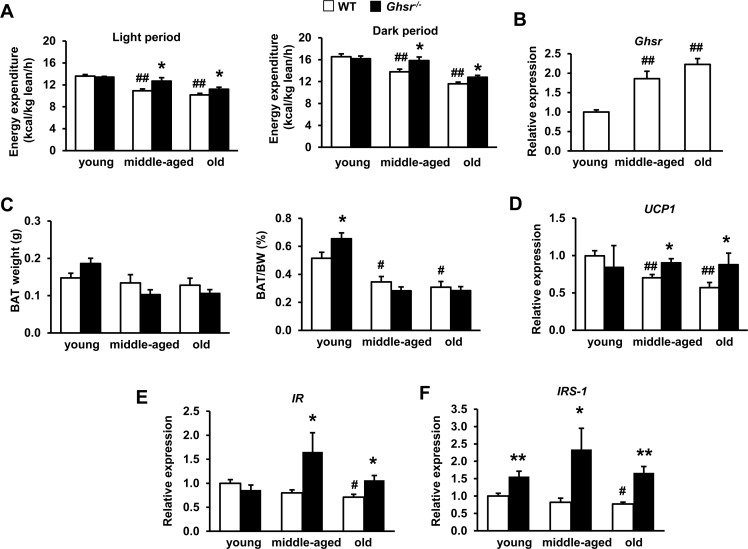
Ghrelin receptor ablation attenuates age-associated decline of thermogenesis and enhances insulin signaling in BAT (**A**) Energy expenditure of young, middle-aged and old WT and *Ghsr^−/−^* mice normalized by lean body mass. (**B**) *Ghsr* expression in BAT of young, middle-aged and old WT mice. (**C**) BAT mass and percentage of BAT weight over body weight in young, middle-aged and old WT and *Ghsr^−/−^* mice. (**D**) *UCP1* expression in BAT of young, middle-aged and old WT and *Ghsr^−/−^* mice. *IR* (**E**) and *IRS-1* (**F**) expression in BAT of young, middle-aged and old WT and *Ghsr^−/−^* mice. Young (4-months), middle-aged (10-months) and old (22-months) mice were used. *N* = 6-14. *p<0.05, **p<0.001, WT vs. *Ghsr^−/−^*; #*p*<0.05, ##*p*<0.001, old or middle-aged vs. young.

Similar to our previous reports [34, 45], energy expenditure of WT mice decreased with age in both light and dark cycles, and GHS-R ablation increased energy expenditure in middle-aged and old mice (Fig. 3A). GHS-R ablation did not alter food intake or physical activity in any age group, indicating that GHS-R exerts its effects primarily by regulating energy expenditure. We studied GHS-R expression in BAT of young, middle-aged and old mice, and found that *Ghsr* expression in BAT increased during aging (Fig. 3B).

Higher circulating ghrelin and increased GHS-R expression in BAT of aged mice suggest enhanced ghrelin signaling in BAT during aging, which may promote age-associated decline of thermogenesis. To further define the role of GHS-R in thermogenesis of BAT, we compared BAT mass and *UCP1* expression in BAT of young, middle-aged and old WT and *Ghsr^−/−^* mice. Although the absolute weight of BAT didn't change with age, BAT mass normalized to body weight decreased during aging in both WT and *Ghsr^−/−^* mice (Fig. 3C). There was no significant difference in the ratio of BAT**/**body weight between WT and *Ghsr^−/−^* mice in middle-aged and old mice, while the ratio of BAT**/**body weight in young *Ghsr^−/−^* mice was higher than that of young WT mice (Fig. 3C). While *UCP1* expression in BAT of WT mice declined during aging, higher *UCP1* expression was detected in BAT of GHS-R ablated aged (middle-aged and old) mice, maintaining expression levels similar to that of young mice (Fig. 3D). These results suggest that GHS-R ablation improves thermogenesis through regulation of BAT activity, but not BAT mass.

It was reported that BAT regulates glucose homeostasis and insulin sensitivity [8, 9]. It has been shown that insulin signaling in BAT is activated by cold stress [48]. Our previous studies showed that aged *Ghsr^−/−^* mice have improved insulin sensitivity [34]. Indeed, we detected lower expression of insulin receptor (*IR*) and insulin receptor substrate 1 (*IRS-1*) in BAT of old WT mice, but GHS-R ablation increased *IR* and *IRS-1* expression in BAT (Fig. 3E and 3F). Together, these data suggest that GHS-R expression in BAT is increased during aging, and ablation of GHS-R attenuates age-associated decline of thermogenesis and insulin sensitivity in BAT.

### Ablation of GHS-R activates thermogenic signaling and improves mitochondrial dynamics of BAT

**Figure 4:**
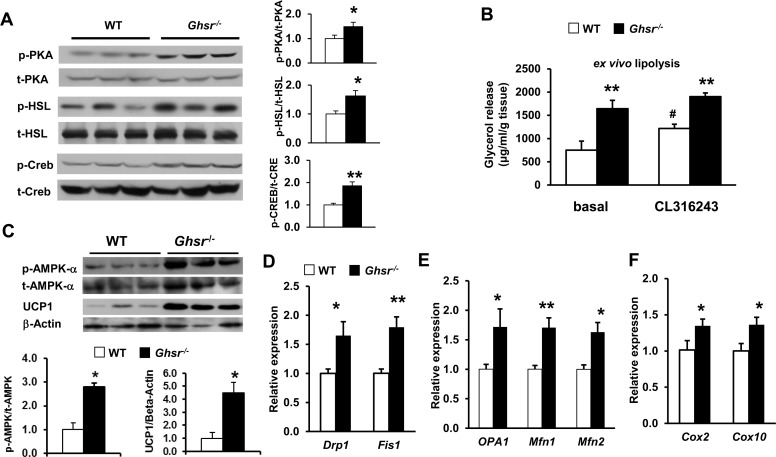
Ablation of GHS-R activates thermogenic signaling cascade and improves mitochondrial dynamics of BAT in aged mice The representative data presented here were from 22 month-old WT and *Ghsr^−/−^* mice. (**A**) Representative Western blots of key thermogenic regulators in BAT of aged WT and *Ghsr^−/−^* mice. (**B**) *Ex vivo* lipolysis of BAT of aged WT and *Ghsr^−/−^* mice, treated with or without 10 μM CL316243. (**C**) Representative Western blots show phosphorylated AMPK α (p-AMPK-α), total AMPK (t-AMPK-α) and UCP1 protein expression in BAT of old WT and *Ghsr^−/−^* mice after 4 hours of cold exposure. (**D**) Expression of mitochondrial fission genes in BAT of aged WT and *Ghsr^−/−^* mice. (**E**) Expression of mitochondrial fusion genes in BAT of aged WT and *Ghsr^−/−^* mice. (**F**) Expression of mitochondrial complex biogenesis markers in BAT of aged WT and *Ghsr^−/−^* mice. *N* = 6. *p<0.05, **p<0.001, WT vs. *Ghsr^−/−^*; #*p*<0.05, basal vs. CL316243 treatment.

BAT thermogenic capacity is determined by the availability of fuel substrate in BAT and UCP1 activity in mitochondria [3]. Protein kinase A (PKA) activates hormone-sensitive lipase (HSL), which is a predominant lipase for catecholamine-stimulated lipolysis in brown adipocytes [49]. Our data revealed that ablation of GHS-R stimulates PKA and HSL activation in BAT, indicative of stimulated thermogenic signaling (Fig. 4A). cAMP response element-binding protein (Creb) is a known downstream target of PKA; phosphorylation of Creb is known to increase the expression of mitochondrial respiratory chain proteins and UCP1 expression [50, 51]. As expected, Creb phosphorylation is increased in BAT of *Ghsr^−/−^* mice (Fig. 4A). Consistently, our *ex vivo* lipolysis study showed that β-adrenergic agonist CL316243-treated brown adipocytes of *Ghsr^−/−^* mice exhibited higher basal and stimulated glycerol release when compared with those of WT mice (Fig. 4B). These data together demonstrate that GHS-R ablation activates thermogenic signaling cascade and promotes lipolysis in BAT.

The 5′ AMP-activated protein kinase (AMPK), a key nutrient and energy sensor, is the master regulator of cellular energy metabolism in many tissues, including BAT [52, 53]. Cold exposure increases AMPK activity [52], and aging is associated with decreased AMPK activity [54]. To test whether GHS-R ablation in BAT activates AMPK, we investigated AMPK activity by studying protein levels of phosphorylated AMPK and total AMPK. Enhanced phosphorylated AMPK was detected in BAT of *Ghsr^−/−^* mice, and was correlated with increased UCP1 (Fig. 4C). This result suggests that AMPK activity may facilitate GHS-R mediated thermogenic regulation.

Mitochondrial biogenesis and dynamics are critical for mitochondrial function [20], and mitochondrial dynamics have been suggested to play a critical role in the pathogenies of insulin resistance [55-57]. We thus studied expression of key mitochondrial dynamics genes in BAT of aged WT and *Ghsr^−/−^* mice. The expression of *Drp1*, *Fis1*, *OPA1*, *Mfn1*, and *Mfn2* was significantly increased in aged *Ghsr^−/−^* mice (Fig. 4D and 4E). Consistently, the expression of subunits of mitochondrial respiratory chain complexes IV, *cox2* and *cox10* was increased in the BAT of *Ghsr^−/−^* mice, indicative of increased mitochondrial activity (Fig. 4F). These data suggest that mitochondrial dynamics may also play a role in GHS-R mediated thermogenic regulation.

### GHS-R regulates BAT thermogenesis through both central and peripheral mechanisms

**Figure 5:**
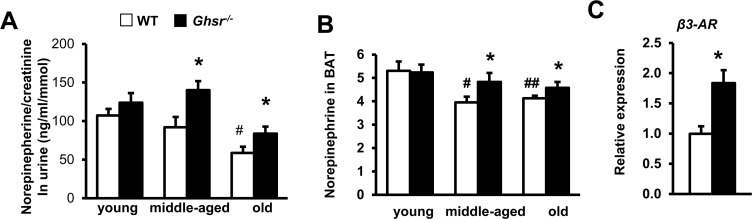
Ablation of GHS-R prevents age-associated decline of norepinephrine (NE) and enhances NE-responsiveness NE concentrations in the urine (**A**) and NE levels in BAT (**B**) of young, middle-aged and old WT and *Ghsr^−/−^* mice. Young (4-months), middle-aged (10-months) and old (22-months) mice were used. (**C**) *β3-*AR mRNA expression in BAT of 22 month-old WT and *Ghsr^−/−^* mice. *N* = 6-10. *p<0.05, WT vs. *Ghsr^−/−^*; #*p*<0.05, ##*p*<0.001, old or middle-aged vs. young.

We have evidence that GHS-R regulates thermogenic function, and ablation of GHS-R attenuates the age-associated decline of thermogenesis [34]. However, since the data were obtained from global GHS-R knockout mice, we could not determine with certainty whether the effect of GHS-R on thermogenesis is mediated through central SNS and/or peripheral brown adipocytes. SNS activation increases catecholamine (norepinephrine, NE) release at SNS endings in BAT, which in turn activates β3-AR in brown adipocytes. To determine the role of SNS-induced catecholamine release in GHS-R mediated thermogenesis, we assessed NE in the circulation and in BAT of WT and *Ghsr^−/−^* mice. Since urinary cate-cholamines are less likely to be affected by handling stress [58], we measured NE concentration in urine of young, middle-aged and old WT and *Ghsr*^−/−^ mice. As expected, urinary NE decreased with age in WT mice, while GHS-R ablated mice maintained NE at a level comparable to that of young mice (Fig. 5A).

Similarly, NE content in BAT of WT mice declined with age, while GHS-R ablation prevented NE decline in BAT of middle-age and old mice (Fig. 5B). Consistently, the expression of *β3-AR* in BAT was higher in old *Ghsr^−/−^* mice than in old WT mice (Fig. 5C). Increased SNS-induced NE release and *β3-AR* expression in BAT of old *Ghsr*^−/−^ mice suggest that GHS-R ablation regulates BAT thermogenesis, at least in part, by activating central SNS-mediated thermogenic signaling pathway of SNS-NE-β3-AR.

**Figure 6:**
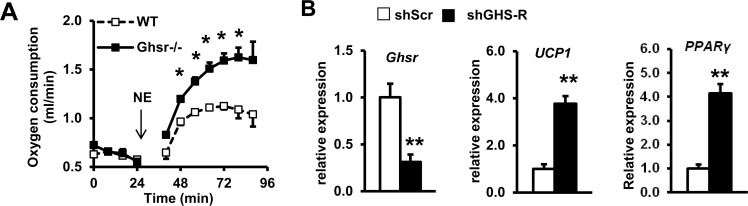
GHS-R directly regulates thermogenic activation in brown adipocytes (**A**) Oxygen consumption of old WT and *Ghsr^−/−^* mice after 1.5 mg/Kg subcutaneous injection of NE. *N* = 10-12. *p<0.05, WT vs. *Ghsr^−/−^*. (**B**) Expression of *Ghsr*, *UCP1* and *PPARγ* in shGHS-R knockdown HIB1B cells. shScr represents scramble shRNA; shGHS-R represents shRNA specific for GHS-R. *N* = 9. **p<0.001, shScr vs. shGHS-R.

To determine NE sensitivity of BAT, we studied NE-induced thermogenesis in old WT and *Ghsr^−/−^* mice. O_2_ consumption after NE injection was significantly higher in old *Ghsr^−/−^* compared with old WT mice (Fig. 6A), suggesting that BAT of old *Ghsr^−/−^* mice is more sensitive to NE stimulation. To assess whether GHS-R has direct effect in brown adipocytes, we further knocked down GHS-R in brown adipocytes (HIB1B) using shGHS-R. Transfection with shGHS-R resulted in 70% knockdown of GHS-R compared with scrambled (shScr) control (Fig. 6B). The expression of thermogenic marker *UCP1* and adipogenic marker Peroxisome Proliferator-Activated Receptor γ (*PPARγ*) was significantly increased in isoproterenol-stimulated GHS-R knockdown HIB1B cells (Fig. 6B). These data suggest that GHS-R also directly inhibits thermogenic activity in brown adipocytes.

## DISCUSSION

We previously showed that ghrelin decreases *UCP1* expression in brown adipocyte HIB1B cells through GHS-R [34]. Current data suggest that obestatin increases *UCP1* expression in HIB1B cells, while DAG has no effect. Thus, ghrelin and obestatin exert opposing effects on thermogenesis. Our data are in agreement with published reports showing that ghrelin decreases *UCP1* expression in BAT [59], while obestatin attenuates the hypothermia of *Ghrelin^−/−^* mice [60]. We observed improved thermogenesis in *Ghsr^−/−^* mice, but not in *Ghrelin^−/−^* mice [34, 45]. The absence of a thermogenic phenotype in *Ghrelin^−/−^* mice may be explained by the fact that both ghrelin and obestatin are absent in *Ghrelin^−/−^* mice; the opposing thermogenic effects of ghrelin and obestatin are eliminated, thus showing no thermogenic phenotype. On the other hand, *Ghsr^−/−^* mice express both ghrelin and obestatin. Since GHS-R is exclusively activated by ghrelin, only ghrelin signaling is blocked in *Ghsr^−/−^* mice, while obestatin signaling is intact. In *Ghsr^−/−^* mice, the stimulatory effect of obestatin on thermogenesis is unopposed, which may explain the increased thermogenesis observed in *Ghsr^−/−^* mice. Thus, the differential thermogenic phenotypes of *Ghrelin^−/−^* and *Ghsr^−/−^* mice are likely due to the opposing effects of ghrelin and obestatin on thermogenesis.

Fasting is associated with decreased energy expenditure, and UCP1 expression in brown fat decreases during fasting [61, 62]. Fasting is associated with increased ghrelin and decreased obestatin in the circulation [26, 41]. The changes of ghrelin and obestatin during fasting may contribute to the decrease of thermogenesis and energy expenditure. After meals, postprandial ghrelin and obestatin decrease, the ratio of ghrelin and obestatin may be involved in the regulation of diet-induced thermogenesis. Aging is associated with severely diminished thermogenic function [10, 11]. Here we found that circulating ghrelin is increased during aging. We previously reported that low levels of GHS-R expression are detectable in BAT [34]. Current data reveal GHS-R expression in BAT is increased during aging. Higher concentration of ghrelin has been shown to be associated with lower resting metabolic rate and postprandial thermogenesis in humans [63]. Ghrelin promotes fat deposition in white fat and suppresses sympathetic nerve activity in brown fat [64, 65]. Our new data show that thermogenic suppressor ghrelin increases during aging; conversely, thermogenic stimulator obestatin remains steady during aging, which may contribute to the age-associated decline of thermogenesis. Collectively, our data suggest that ghrelin signaling contributes to the age-associated thermogenic dysfunction, which in turn promotes obesity and insulin resistance in aging.

Our data further demonstrate that GHS-R regulates thermogenic signaling cascades in BAT. GHS-R ablation activates thermogenic signaling pathway PKA-Creb-UCP1, and lipolytic pathway PKA-HSL-UCP1. It has been reported that insulin resistance in BAT is associated with thermogenic defect in obese and diabetic rats [48, 66]. Age-associated mito-chondrial dysfunction is a well-known cause for insulin resistance [15-17]. It has been shown that acute cold exposure improves glucose clearance and insulin sensitivity of BAT by activating the PKA-HSL-Lipolysis pathway [48]. We observed decreased expression of *IR* and *IRS-1* in BAT of old *Ghsr^−/−^* mice, suggesting that GHS-R ablation improves insulin sensitivity of BAT, which then further promotes BAT thermogenic activation.

AMPK is the master regulator of cellular energy metabolism in BAT [52, 53]. It is well documented that activation of AMPK enhances PGC1α-dependent transcription, which senses energy availability and stimulates mitochondrial DNA and protein synthesis [53, 67]. Ghrelin has been shown to have differential effects on AMPK activity: stimulatory in hypothalamus and heart, but inhibitory in liver and WAT [68, 69]. Our previous study showed that GHS-R ablation increases mitochondrial content [34]. Our current data reveal that higher levels of phosphorylated AMPK in BAT of *Ghsr^−/−^* mice are correlated with increased UCP1. This suggests that GHS-R ablation may activate AMPK to increase mitochondrial biosynthesis of BAT, thus enhancing thermogenesis.

Mitochondrial dynamics play a crucial role in mitochondrial function [20]. Abnormal mitochondrial structure has been observed i n obese and type 2 diabetic patients [22]. GHS-R deletion increased the expression of both fusion- and fission-related genes in BAT of aged *Ghsr^−/−^* mice, indicative of an active state of mitochondrial dynamics and improved mitochondrial homeostasis. Improved mitochondrial homeostasis will enhance the sensitivity of mitochondria to FFA to further promote mitochondrial uncoupling. The data suggest that suppressing ghrelin signaling preserves youthful mitochondrial dynamics in BAT during aging. It has been shown that obesity induces *Mfn2* deficiency which results in reduced mitochondrial activity, and *Drp1* deletion leads to reduction of mtDNA content [24, 70]. We found that the expression of *Mfn2* and *Drp1* was increased in BAT of aged *Ghsr^−/−^* mice, consistent with the increased thermogenic activity and mtDNA content detected in BAT of aged *Ghsr^−/−^* mice [34]. In addition, the subunits of mitochondrial respiratory chain complexes IV, such as mtDNA-encoded cox2 and nuclear DNA-encoded cox10, were also increased in *Ghsr^−/−^* mice, lending further support to the increased mitochondrial biogenesis. Taken together, we conclude that GHS-R ablation activates thermogenic signaling cascades and promotes lipolysis in BAT; GHS-R ablation enhances mitochondrial function of BAT by increasing mitochondrial biogenesis and stimulating mitochondrial dynamics.

Last, we explore the potential site(s) of action of GHS-R mediated thermogenesis. The phenotype we observed in *Ghsr^−/−^* mice could result from GHS-R mediated effects in central and/or peripheral sites. Thyroid hormones are important regulators of thermogenesis [71]. However, serum T3 and T4 concentrations were comparable in WT and *Ghsr^−/−^* mice [34], indicating that the elevated thermogenesis observed in old *Ghsr^−/−^* mice is not due to changes in circulating thyroid hormones. Sympathetic activity is known to play a dominant role in thermogenic regulation [3, 4]. Aging is associated with decreased stress responsiveness, because of reduced circulating NE and impaired hypothalamic-pituitary-adrenal axis [72]. In the current study, we found that GHS-R ablation prevents age-associated decrease of NE in the circulation, suggesting that GHS-R mediated thermogenesis is likely regulated centrally via SNS-induced NE release. On the other hand, our data also showed that GHS-R ablation increases NE-induced O_2_ consumption, indicative that BAT of *Ghsr^−/−^* mice has increased sensitivity to NE stimulation; this suggests that GHS-R ablation may directly affect the thermogenic response of BAT. Indeed, we found that knockdown of GHS-R in HIB1B cells up-regulates the thermogenic mediator UCP1, suggesting that ghrelin signaling regulates thermogenesis directly in BAT. Together, our data suggest that ghrelin signaling regulates BAT ther-mogenesis via both central and peripheral mechanisms.

**Figure 7:**
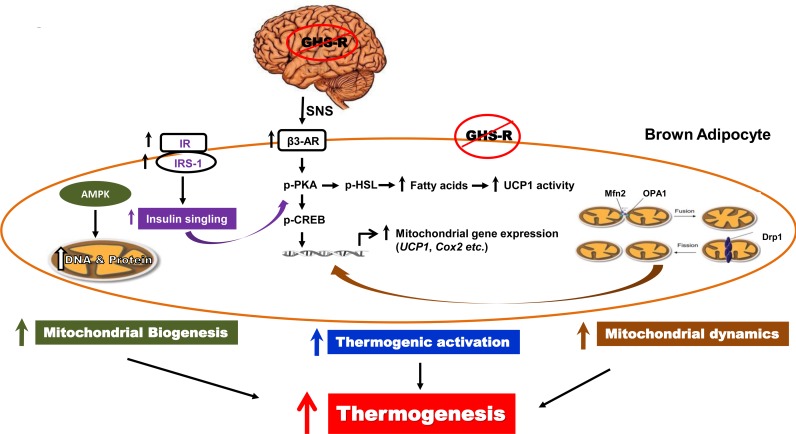
Schematic diagram of GHS-R mediated thermogenic regulation in brown adipocytes Our data suggest that ghrelin signaling may regulate thermogenesis in BAT via the following 4 independent and interconnected signaling pathways: 1) Ablation of GHS-R stimulates SNS-mediated NE release, which in turn induces β3-AR expression, subsequently activating thermogenic signaling cascades in BAT. This involves activation of thermogenic signaling pathway PKA-CREB-UCP1 and lipolytic pathway PKA-HSL-UCP1. 2) Ablation of GHS-R enhances insulin signaling in BAT, which improves insulin sensitivity of BAT and activates key thermogenic regulator PKA. 3) Ablation of GHS-R enhances AMPK activity in BAT, which increases DNA and protein synthesis of mitochondria, thus increasing mitochondrial biogenesis. 4) Ablation of GHS-R augments mitochondrial dynamics, enhancing both mitochondrial fission and fussion; this restores mitochondrial architecture and improves mitochondrial homeostasis. Improved mitochondrial homeostasis enhances the sensitivity of mitochondria to FFA to further promote mitochondrial uncoupling. Collectively, GHS-R ablation increases thermogenesis in BAT by activating thermogenic signaling, sensitizing insulin signaling, increasing mitochondrial biogenesis, and enhancing mitochondrial dynamics.

In summary, our studies show that ghrelin and GHS-R are important thermogenic regulators, and increased ghrelin signaling in BAT during aging contributes to the age-associated thermogenic impairment. GHS-R mediated thermogenesis in BAT is attributable to thermogenic signaling activation, insulin sensitivity, mitochondrial biogenesis and dynamics of BAT (Fig. 7). GHS-R likely regulates thermogenesis via both central mechanisms involving SNS-induced NE release and direct effect in BAT. Thus, GHS-R antagonists may serve as a unique class of drugs that can prevent/treat age-associated obesity and insulin resistance by enhancing thermogenesis.

## METHODS

### Animals

*Ghsr^−/−^* mice were generated and genotyped as described previously [29]. All mice used in the experiments are male mice, which have been backcrossed 13 generations onto C57BL/6J background. Wild-type (WT) and homozygous knockout mice (*Ghsr^−/−^*) were housed and bred in a pathogen-free facility at Baylor College of Medicine. Animals were housed under controlled temperature and lighting cycle (75±1 °F; 12h light-dark cycle) with free access to regular chow and water. All experiments were approved by the Animal Care Research Committee of Baylor College of Medicine. To determine data-relevant age cohorts, we tested young mice at 3-4 months-of-age, middle-aged mice at 10-12 months-of-age, and old mice at 18-24 months-of-age. “Aged” is defined as middle-aged or old mice.

### Real-time RT-PCR

Total cellular RNA was isolated using TRIzol Reagent (Invitrogen, Carlsbad, CA), following the manufacturer's instructions. In order to remove potential genomic DNA contamination, RNA was treated with DNAse and run on gels to validate the purity and quality. cDNA was synthesized from 1 μg RNA using the SuperScript III First-Strand Synthesis System (Invitrogen, Carlsbad, CA). Real-time RT-PCR was performed on an ABI 7900 using the SYBR Green PCR Master Mix, the Taqman gene expression Master Mix (Applied Biosystems, Carlsbad, CA), or Bio-Rad using iQ SYBR GREEN supermix (Bio-Rad, Hercules, CA), according to the protocols provided by the manufacturers. *18s RNA* and *β-actin* were used as internal controls. All primer and probe information is available upon request.

### Plasma analyses

For active ghrelin and des-acyl ghrelin (DAG): about 200 μl blood was collected in EDTA-coated capillary tubes with or without 18 hours fasting. After spin down, plasma was transferred to an ice-cold tube with 5 μl 1N HCl. Then, 1 μl fresh PMSF (10 mg/ml in methanol, Sigma, St. Louis, MO) was added and gently mixed. 25 μl plasma was measured with either RIA assay kits (EMD Millipore, Billerica, MA) or ELISA kits (Mitsubishi Kagaku Iatron, Tokyo, Japan). For obestatin: about 200 μl blood was collected in EDTA-coated capillary tubes. The blood sample was then transferred to tubes and mixed with 20 μl aprotinin (6 TIU/ml, Phoenix, Milpitas, CA). After spin down, plasma was transferred to a new ice-chilled tube. 50 μl of plasma was measured with an EIA kit (Phoenix, Milpitas, CA). For insulin: about 200 μl blood was collected in EDTA-coated capillary tubes. The blood sample was then transferred to tubes. After spin down, plasma was transferred to a new ice-chilled tube. 50 μl of plasma was measured with a RIA kit (Linco Research, St. Charles, MI), following manufacture's instruction.

### Metabolic characterizations

Metabolic parameters were obtained using an Oxymax open-circuit indirect calorimetry system (Columbus Instruments, Columbus, OH), as previously described [34, 73]. Briefly, mice were individually caged in chambers and given free access to regular chow and water for one week prior to tests. The first 24 hours in calorimetry chambers was considered the acclimation phase, and data were analyzed only for the following 48 hours. Oxygen consumption (VO_2_) and carbon dioxide production (VCO_2_) by each animal were measured. Energy expenditure (EE, or heat generation) was calculated from VO_2_ and VCO_2_ gas exchange data as follows: EE = (3.815+1.232×VCO_2_/VO_2_) × VO_2_. Energy expenditure was then normalized to lean body mass.

For norepinephrine-induced thermogenesis, the animals were anesthetized with pentobarbital (90 mg/kg, i.p.), and indirect calorimetry was performed for 30 minutes to obtain basal values as earlier described [74]. The individual mouse was then briefly removed from the calorimetry chambers, injected with norepinephrine (1.5 mg norepinephrine/kg, subcutaneously), and then returned to the metabolic chamber, and oxygen consumption was then measured for another 60 minutes.

### Urinary norepinephrine assay

The mice urinary NE concentration was determined as described previously [58, 73]. Briefly, mice were grabbed quickly to induce urination. About 200 μl urine sample was collected into a tube with 2 μl 6N HCl, to determine NE level using ELISA assay (IBL Inc., Minneapolis, MN). Meanwhile, about 10 μl urine sample was transferred to another tube for creatinine determination using ELISA assay (Quidel Corporation, San Diego, CA). Creatinine-normalized norepinephrine levels are shown.

### BAT norepinephrine assay

The BAT NE concentration of the mice was determined as previously described [75]. Briefly, BATs were homogenized by sonication in homogenization buffer (1 N HCl, 0.25 M EDTA, 1 mM Na_2_S_2_O_5_). Cell debris was then pelleted by centrifugation at 13,000 r.p.m. for 15 min at 4 °C. The cleared homogenates were collected and stored at −80 °C before quantification. Fifty μl tissue lysate was used for measurement of NE using ELISA assay (Rocky Mountain Diagnostics, Colorado Springs, CO) following the manufacturer's protocol. All samples were normalized to total tissue protein content.

### Western blot analyses

Brown adipose tissues were lysed in RIPA buffer with Complete Protease Inhibitor Cocktail (Roche Inc., Indianapolis, IN). Protein concentration was determined with BCA protein assay kit (Pierce, Rockford, IL). Twenty microgram of protein of each sample was separated by SDS-PAGE, and electro-transferred to nitrocellulose membrane for immunoblot analyses. The following antibodies were used: anti-p-PKA (Tyr197) (Cell Signaling, Danvers, MA, 4781S, 1:1000), anti-t-PKA (Cell Signaling, Danvers, MA, 4782, 1:1000), anti-p-HSL (Ser563) (Cell Signaling, Danvers, MA, 4139S, 1:1000), anti-t-HSL (Cell Signaling, Danvers, MA, 4107, 1:1000), anti-p-Creb (Ser133) (Cell Signaling, Danvers, MA, 9191S, 1:1000), anti-t-Creb (Cell Signaling, Danvers, MA, 9197, 1:1000), p-AMPKα (T172) (Cell Signaling, Danvers, MA, 2535L, 1:1000), anti t-AMPK (Cell Signaling, Danvers, MA, 2603S, 1:1000), anti-*UCP1* (ABCAM, Cambridge, MA Ab10983, 1:10000), anti-β-actin (Cell Signaling, Danvers, MA, 4967S, 1:1000), HRP-conjugated anti-mouse (GE Healthcare UK Limited, 1:10,000), and anti-rabbit (GE Healthcare UK Limited, 1:10,000). The SuperSignal West Pico Chemiluminescent kit (Pierce, Rockford, IL) was used as substrates.

### *Ex vivo* lipolysis assay

The lipolysis activity of BAT was measured, using *ex vivo* lipolysis assay as described [76]. Briefly, interscapular BAT was dissected and separated into two pieces. Each piece was put into culture medium {DMEM with 0.5% fatty acid free BSA (Sigma, St. Louis, MO)} and minced into tiny pieces with scissors. The tissues were incubated at 37°C with 10 μM CL316243 (Sigma, St. Louis, MO) as stimulated condition, or DMSO as basal condition. 2 hours later, medium was collected and heated at 85°C for 10 minutes. After spin down, clear supernatant was transferred to a new tube, and 10 μl was used to measure free glycerol content using Free Glycerol Reagent (Sigma, St. Louis, MO). Lipolysis activity was represented by glycerol concentrations, normalized by weight of the tissue.

### HIB1B cell culture

HIB1B pre-adipocytes were cultured in Dulbecco's modified Eagle's medium (DMEM) containing 10% bovine calf serum. At confluence, HIB1B cells were induced to differentiate for 3 days in DMEM with 10% Cosmic Calf Serum (Hyclone, Logan, UT), supplemented with 5 μg/ml insulin (Sigma, St. Louis, MO), 0.5 mM isobutylmethylxanthine (Sigma, St. Louis, MO), 1 μM dexamethasone (Sigma, St. Louis, MO), and 1 nM triiodothyronine (Sigma, St. Louis, MO). The cells were then fed every 2 days with 10% Cosmic Calf Serum in DMEM, containing only insulin and triiodothyronine, at the concentrations mentioned above. On day 6 of differentiation, cells were treated with different concentrations of ghrelin, DAG, obestatin or saline for 24h. Cells were stimulated with 1 μM isoproterenol (Sigma, St. Louis, MO) for 6h prior to harvest.

### Generation of GHS-R knockdown HIB1B cell line

The predesigned GHS-R shRNA (TG506242) and scrambled shRNA (GCACTACCAGAGCTAACTCAGATAGTACT) in pGFP-V-RS vector were purchased from Origene (Rockville, MD). The shRNAs were transfected into HIB1B cells by Lipofectamine® 2000™ (Invitrogen, Carlsbad, CA), according to the manufacturer's instruction. Briefly, 1 million HIB1B cells were seeded in a 10 cm dish for 12 hours. 2 hours before transfection, medium was changed. Five μg shRNA plasmids were mixed with Lipofectamine® 2000™ reagent and kept at room temperature for 5 minutes. Then, shRNA mixture was added to the dishes. 12 hours later, shRNA mixture was removed, and fresh medium was added. Then 24 hours later, 1 μg/ml puromycin was added to the medium to select positive knockdown cells. After 8 days of selection, all positive cells were pooled together as a stable cell line.

### Statistics

Two-factor ANOVA was used to evaluate the significance of interaction between genotype and age, and *post hoc* test was used to follow up the significant differences between ages and genotypes. Data are represented as mean ± SEM, and statistical significance is set to a minimum of *p*<0.05.
